# Association between Chronic Environmental Lead (Pb) Exposure and Cytokines in Males and Females of Reproductive Age from Kabwe, Zambia

**DOI:** 10.3390/ijerph20085596

**Published:** 2023-04-20

**Authors:** Andrew Kataba, Yared Beyene Yohannes, Hokuto Nakata, John Yabe, Haruya Toyomaki, Kaampwe Muzandu, Golden Zyambo, Yoshinori Ikenaka, Kennedy Choongo, Mayumi Ishizuka, Shouta M. M. Nakayama

**Affiliations:** 1 Laboratory of Toxicology, Department of Environmental Veterinary Sciences, Faculty of Veterinary Medicine, Hokkaido University, Kita 18, Nishi 9, Kita-ku, Sapporo 060-0818, Japan; 2 School of Veterinary Medicine, The University of Zambia, Lusaka P.O. Box 32379, Zambia; 3 Department of Chemistry, College of Natural and Computational Science, University of Gondar, Gondar P.O. Box 196, Ethiopia; 4 School of Veterinary Medicine, University of Namibia, Windhoek P/B. 13301, Namibia; 5 Water Research Group, School of Environmental Sciences and Development, North-West University, Potchefstroom 2531, South Africa; 6 Translational Research Unit, Veterinary Teaching Hospital, Faculty of Veterinary Medicine, Hokkaido University, Sapporo 060-0818, Japan; 7 One Health Research Center, Hokkaido University, Sapporo 060-0818, Japan

**Keywords:** lead, chronic, immunomodulatory, cytokines, sex-linked

## Abstract

Lead (Pb) poisoning remains a great public health challenge globally known to induce a wide range of ailments in both children and adults. The current study investigated the association of chronic environmental Pb exposure and immunomodulatory cytokines tumor necrosis factor alpha (TNF-α) and interleukin-8 (IL-8) in adult males and females living in Kabwe, Zambia. The standard human cytokine/chemokine Milliplex assay was used to quantify plasma cytokines from four groups categorized as low (<10 μg/dL) and high (>10 μg/dL) blood lead level (BLL) groups, namely, low BLL female (*n* = 47; BLL = 3.76 μg/dL), low BLL Male (*n* = 43; BLL = 4.13 μg/dL), high BLL female (*n* = 21; BLL = 23.5 μg/dL), and high BLL male (*n* = 18; BLL = 23.7 μg/dL), respectively. The low BLL group was associated with increased TNF-α levels, and the high BLL group was associated with reduced TNF-α levels in female subjects. No associations between BLL and the levels of IL-8 and TNF-α cytokines were observed in either females or males, respectively. A negative correlation between BLL and TNF-α was found in female subjects, suggesting that an increase in BLL accompanied by a reduction in TNF-α. The reduced levels of circulating TNF-α in female subjects suggest that chronic Pb exposure could predispose females to immune and inflammation-related disorders than their male counterparts. Further studies are recommended to ascertain the impact of chronic Pb exposure on immunomodulatory cytokines, especially in females.

## 1. Introduction

Lead (Pb) poisoning remains a great public health challenge globally and accounts for approximately 0.6% of the world’s total disease burden [1]. Despite the advanced knowledge regarding Pb as a toxicant with deleterious effects in animals and humans, Pb’s unique physical and chemical properties have kept it in use at both industrial and domestic levels [2]. Lead naturally occurs in the environment as galena sulphide in very small amounts. Large quantities of environmental Pb are attributed to industrial discharges such as mining or manufacturing processes including lead battery recycling [3]. Although there is a global decline in Pb usage due to its known toxic effects, occupational and environmental Pb exposure remains a problem in both developed and developing countries [4].

Lead poisoning may take two forms, namely, acute and chronic poisoning with the former being less common than the latter [5]. Acute Pb poisoning is linked to accidental or occupational Pb exposure and is characterized by blood lead levels (BLLs) between 100–120 μg/dL with a rapid onset of clinical signs such as constipation, abdominal pain, headaches, emesis, seizures, coma, and death [6,7,8]. The clinical manifestations of chronic Pb poisoning may include recurrent and persistent symptoms that accompany acute Pb poisoning with relatively lower BLLs compared to typical acute Pb-related clinical manifestations [6]. In addition, neurobehavioral impairments with behavioral and learning deficits as sequelae even at low chronic Pb exposure are common in children [9].

On the other hand, chronic Pb poisoning in adults has been reported to elicit personality changes and other maladies including cardiovascular, hematological, and renal-related diseases [5]. Moreover, in both adult males and females, chronic Pb poisoning is known to cause insidious reproductive and immune-related derangements [10]. In males, BLLs exceeding 40 µg/dL have been reported to be associated with low sperm count, poor sperm morphology and motility, and a reduction in the level of serum testosterone [9,11]. Lead-induced reproductive disorders in females such as miscarriage, low birth weight, and pre-term delivery have been reported in moderately low BLLs less than or equal to 30 µg/dL [12]. Whereas the mechanisms of Pb-induced reproductive disorders are complex, Pb-induced disruption of the antioxidant system, steroidogenesis, and immunomodulatory factors such as cytokines, among others, have been implicated [2,5,13]. Cytokine levels may be directly or indirectly altered by Pb exposure. In particular, the effect of Pb exposure on cytokines such as tumor necrosis factor-alpha (TNF-α) and interleukin-8 (IL-8) is linked to infertility and cardiomyopathies in adults [14]. Cytokines are small peptides found in cells whose role as regulators of innate and adaptive immune responses is well recognized [15]. These immunomodulatory cytokines play a vital role in combating both communicable and non-communicable diseases [16]. Hence, dysregulation of cytokine levels exacerbates ailments by enhancing pathological processes such as autoimmune maladies, cancer progression, and idiopathic cardiac diseases [17].

A classical scenario of chronic Pb poisoning in males and females of reproductive age has been reported in Kabwe, Zambia [18]. Based on the recent report by Yabe et al. [18], over 50% of both men and women of reproductive age had BLLs that ranged between 5 and 44 µg/dL. Despite the reported BLL levels, the impact of chronic Pb poisoning in adults of reproductive age in Kabwe remains unknown. Hence, the objective of the current study was to investigate the association of chronic Pb exposure and immunomodulatory cytokines in adult males and female from Kabwe, Zambia. To the best of our knowledge, the findings reported in the current study form baseline data for immunomodulatory cytokines in chronically environmentally Pb-exposed Kabwe residents.

## 2. Materials and Methods

### 2.1. Study Target Population

The study targeted adult males and females of reproductive ages from Kabwe town in Zambia. Huge piles of Pb contaminated soils dispersed by natural elements such as wind and water runoffs from the abandoned tailings and mine dumps have extensively polluted the town with Pb [19]. In the current study, plasma samples were obtained from blood samples that were collected from adult female and male participants in Kabwe in July and August 2017 as was previously reported by Yabe et al. [18]. The study population was drawn from a wider section of Kabwe, as shown in the map from our previous research [18].

### 2.2. Sampling Strategy

The plasma samples used in this study were randomly drawn based on the BLLs, age, and sex clustering. The BLLs data used were based on the venous blood samples of adult Kabwe residents (male and female) sampled during the July–August 2017 KAMPAI human broad survey that had been previously analyzed using an inductively coupled plasma mass spectrometry (ICP-MS 7700 series; Agilent Technologies, Tokyo, Japan) with a limit of detection of 0.0001 μg/dL, courtesy of Yamada et al. [20]. The quality assurance for the BLLs was performed using certified blood reference material Seronorm^TM^ Trace Elements Whole Blood L-2 (Sero, Billingstad, Norway) with recoveries of 95–105%. The sampling during the survey was approved by the University of Zambia Biomedical Research Ethics Committee (UNZABREC; approval No. 012-04-16), and all participants were volunteers that gave written informed consent. The sex, age, body mass index (BMI), and smoking status were extracted from the questionnaire and screening information that were recorded from participants during the survey. Only healthy males and females of reproductive age of 18 years and above and with no history of smoking were included in this study. Based on the Kabwe residents’ BLLs characteristics, the BLLs were divided into two groups, namely, low BLL and high BLL. The low BLL group represented the low exposure threshold in adults of less than 10 µg/dL, while BLLs above 10 µg/dL were classified as the high BLL group [21]. As shown in Table 1, the BLL means for the low BLL group were 3.76 and 4.13 µg/dL for female and male participants, respectively. For the high BLL group, the BLL mean values were 23.5 µg/dL and 23.7 µg/dL for the female and male groups, respectively. A total of 129 plasma samples (low BLL female: *n* = 47; low BLL male: *n* = 43; high BLL female: *n* = 21; and high BLL male: *n* = 18) were used for cytokine analysis, as shown in Table 1. The BMI and age characteristics were as listed in Table 1.

### 2.3. Cytokine Assay

Based on unpublished pre-experiment cytokine assays we conducted on several cytokines (epidermal growth factor, transforming growth factor alpha, interferon-gamma, interleukin-10, interleukin-15, interleukin-2, interleukin-4, interleukin-6, interleukin-7, interleukin-8, interleukin-1 beta, tumor necrosis alpha, and vascular endothelial growth factor), only interleukin-8 and tumor necrosis factor-alpha were consistently detected in Kabwe human samples. Therefore, only TNF-α and IL-8 were assayed for the current study. The two plasma cytokines were assayed using MILLIPLEX^®^ MAP Kit-Human Cytokine/Chemokine Magnetic Bead Panel (HCYTMAG-60K) (Merck KGaA, Darmstadt, Germany) with strict adherence to the manufacturer’s instructions. All reagents and standard solutions (3.2 to 2000 pg/mL) were stored and prepared for the assays according to the guidelines outlined in the manufacturer’s manual. The frozen plasma samples that were kept at −20 °C were thawed prior to analysis at room temperature. As an initial step, the 96-well plates were conditioned using 200 μL wash buffer on a plate shaker for 10 min at room temperature. Then, the wash buffer was decanted, and all residual amounts were removed by inverting and tapping onto clean absorbent towels. The standards, as well as the positive and negative control samples, were added to appropriate wells, followed by the addition of 25 μL assay buffer to the background and sample wells, respectively. The plasma samples (25 μL) were then added to the sample wells, followed by 25 μL of premixed beads. The plate was then sealed with a plate sealer and wrapped with aluminum foil and incubated with mild agitation overnight for 18 h at 4 °C. Following incubation, the well contents were decanted, and the plate was gently washed with 200 μL two times. The detection antibodies were then added to each well and the plate contents were incubated for 1 h at room temperature. This was followed by the application of the fluorescent conjugate streptavidin-phycoerythrin to each well and incubation for 30 min at room temperature. The well contents were gently decanted, and the plate was washed two times using the wash buffer. Finally, 150 μL sheath fluid was added before running the samples on Luminex^®^ 200^TM^ with xPOTENT^®^ software (Luminex Corporation, Austin, TX, USA) using a standard cytokine protocol.

### 2.4. Data Analysis

The statistical analyses were all performed using GraphPad Prism software (Prism 7 for Windows; Version 5.02, San Diego, CA, USA). The data were reported as mean ± SD (standard deviation). Due to the lack of normality in the data following the application of the Kolmogorov–Smirnov test, the Kruskal–Wallis test and Dunn’s Multiple Comparison test or Mann–Whitney U test were applied to analyze the data. Correlation analyses were performed using the Spearman non-parametric correlation coefficient test. The principal component analysis (PCA) was performed to explore the association between plasma cytokines (TNF-α and IL-8) and age, BMI, and BLL using JMP Pro version 15 (SAS Institute, Cary, NC, USA). All statistical analyses with *p*-value < 0.05 were considered significant.

## 3. Results

### 3.1. BLLs and Demographic Characteristics of the Participants

The general characteristics of the studied subjects are listed in Table 1. In the current study, BLLs were used as the principal determining characteristic for the randomly selected plasma samples and the categorization into low and high BLL groups. The female subjects’ age ranges were 27–69 years for the low BLL and 21–68 years for high BLL group. The mean age between the two groups was not significantly different (*p* = 0.51). The BMI between the low BLL and high BLL groups in females was statistically similar between the two groups. In males, the age range for the low BLL was 19–72 years, and it was 21–66 years in the high BLL group. The mean age between the low BLL and the high BLL, as shown in Table 1, was statistically different (*p* < 0.01). The BMI mean values were not different among the groups.

### 3.2. Plasma TNF-α and IL-8 Cytokines in Female and Male Adults

**Figure 1:**
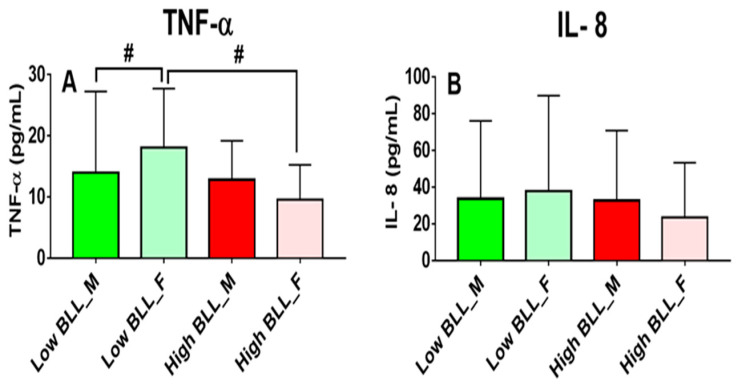
Cytokines in adult female and male plasma samples across the two groups. Key: Low BLL_M = low BLL group in males; Low BLL_F = low BLL group in females; High BLL_M = high BLL group in males; High BLL_F = high BLL group in females (mean ± SD). (**A**) Tumor necrosis alpha (TNF-α) and (**B**) interleukin-8 (IL-8). (# represents *p* < 0.05, Mann–Whitney test).

The plasma cytokine concentration in females ranged between 1.5 and 36.7 pg/mL, with the mean of 17.7 ± 1.45 pg/mL for TNF-α in the low BLL group. In the high BLL group, the TNF-α range was between 1.88 and 39.1 pg/mL with a mean of 11.7 ± 1.80 pg/mL. A statistical significance (*p* < 0.05) reduction in the circulating TNF-α was observed, as shown in Figure 1. A slight but non-significant reduction in IL-8 cytokines levels was observed.

The plasma cytokine levels in male subjects are also shown in Figure 1. The TNF-α was slightly reduced in the high BLL group compared to the low BLL group, with means of 12.6 ± 1.46 pg/mL and 14.6 ± 2.04 pg/mL, respectively. The plasma IL-8 levels were also not statistically different between the two groups.

Following Spearman’s correlation analyses, a positive correlation (r = 0.288, *p* < 0.05) between TNF-α and IL-8 levels was observed in female subjects (Table 2). A non-significant negative correlation (r = −0.183) between age and BLL was observed.

The Spearman’s correlation analyses for the plasma cytokines and other variables in males are shown in Table 3. A non-significant positive correlation between TNF-α and IL-8 was observed. Among the other variables, a positive significant correlation between BLL and age was observed.

### 3.3. Comparative Association between Pb Exposure and Plasma Cytokines in Female and Male Adults

The comparative effect of chronic exposure on plasma cytokines based on sex is shown in Figure 1. The circulating plasma TNF-α cytokine was significantly lower in the low BLL group of the males compared to that of the females in the low BLL group. In the high BLL group, the female TNF-α was slightly lower than that of the male. The IL-8 showed a similar pattern to that of the TNF-α in a non-significant manner.

**Figure 2:**
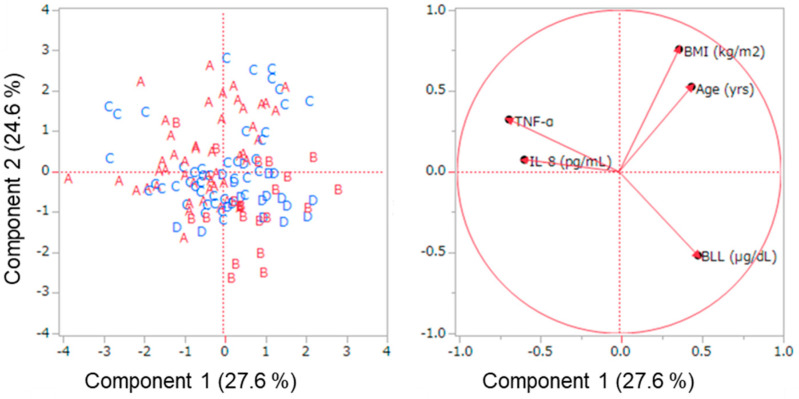
Principal component analysis of the plasma cytokines, tumor necrosis factor alpha (TNF-α) and interleukin-8 (IL-8) and their relationship with the age, body mass index (BMI), and BLL in the total samples analyzed (*n* = 129) in adult females and males (alphabetical letters represents the four groups investigated in the study: A = low BLL female; B = high BLL female; C = low BLL male; and D = high BLL male groups).

Figure 2 shows the PCA results of the male and female plasma TNF-α and IL-8 cytokines and other variables, namely, age, body mass index (BMI), and BLL. The first principal component (PC1) accounted for 27.6%, and PC2 accounted for 24.6% variability of the results (Table S1, Supplementary Materials). The age and BLL were the major contributors to the data variations that were found in PC1, and PC1 had a negative relationship with TNF-α and IL-8 cytokines, as shown in Table S1. Age and BMI accounted for much of the variation in data for PC2, and PC2 had a negative relationship with BLL (Table S1). The overall associations and correlations in the cytokines relative to age, BMI, and BLL as estimated by the Row-wise method are shown in Table S2 (Supplementary Materials). The TNF-α was positively correlated (r = 0.206) with the IL-8. Generally, the cytokine levels reduced with an increase in BLL in both and females. On the other hand, the BLL was negatively correlated (r = −0.210 and r = −0.078) to both TNF-α and IL-8, respectively. No associations were observed between the plasma cytokines, BMI, and age.

## 4. Discussion

In the current study, the first baseline findings that could be associated with chronic environmental Pb exposure on immunomodulatory cytokines, namely, tumor necrosis alpha and interleukin-8 in adult male and female residents of Kabwe, Zambia, are reported. Immunomodulatory cytokines play a vital role in the ability of the body to combat both communicable and non-communicable diseases through adaptive innate immune responses [16]. The dysregulation of cytokine levels therefore can exacerbate ailments by enhancing pathological processes such as autoimmune maladies including cancer progression and cardiomyopathies [17]. Lead-induced toxicities in adult females of reproductive age have been linked to insidious clinical manifestations such as miscellaneous miscarriages, pre-term birth, and babies with low birth weight [9]. Although the mechanisms behind the above clinical manifestations are complex, Pb-induced inflammation and immune system dysregulation have been said to be indirectly or directly linked to immunomodulatory cytokines, in particular TNF-α [22].

In general, the levels of both TNF-α and IL-8 detected in plasma samples of adult males and females of reproductive ages under chronic Pb environmental exposure in our study were similar to the levels that have been reported in occupationally Pb-exposed subjects [13]. The plasma TNF-α levels in both males (low BLL: 12.6 ± 1.46 pg/mL; high BLL: 14.6 ± 2.04 pg/mL) and females (low BLL: 17.7 ± 1.45 pg/mL; high BLL: 11.7 ± 1.80 pg/mL) in the current study were above the normal levels reported in female (0.64 ± 2.37 pg/mL) and male (0.49 ± 2.51 pg/mL) healthy adults, respectively [23]. Moreover, in the current study, environmental Pb exposure’s impact on immunomodulatory cytokines revealed a sex-dependent effect. In particular, the females’ plasma samples revealed an increase in circulating TNF-α cytokines at low BLL (<10 µg/dL) and a reduction in TNF-α levels at high blood lead level (>10 µg/dL) with corresponding levels in interleukin-8. The male plasma cytokines levels showed a similar trend to that observed in the females though not statistically significant.

The differences observed in the levels of the plasma cytokines between males and females have also been reported by other authors who attributed the differences to the inherent differences in the genetic makeup and hormonal profiles [24]. Estradiol hormone has been implicated as one of the factors that accounted for the sex-linked differences in cytokine levels in females and males [25]. In particular, high levels of 17β-estradiol were accompanied by reduced concentrations of TNF-α in peripheral blood cultures [26]. Notwithstanding, in the present study, we observed that female subjects with low BLLs had higher concentrations of TNF-α than those with high BLLs, suggesting that chronic Pb exposure had an association with the concentrations of the immunomodulatory cytokines.

The plasma TNF-α and IL-8 levels were positively correlated in the female subjects in the current study, a pattern that agreed with other authors that demonstrated that an increase in TNF-α was accompanied by a corresponding increase in IL-8 levels [27]. Moreover, the level of IL-8 is dependent on TNF-α, as the latter is known to induce the production of the former in body cells such as macrophages [13]. The relationship between the two cytokines implies that an adverse impact related to Pb exposure through the reduction of the type I helper (Th1) cells that produce TNF-α will eventually impact the other [27].

On the other hand, a negative correlation between BLL and the two immunomodulatory cytokines observed suggests that female Kabwe residents with high BLL will have low levels of these cytokines. Considering the age of female participants in the current study, the findings imply that the deleterious effect of Pb on immunomodulatory cytokines could be among the factors that may negatively impact on fertility and the wellbeing of the fetuses in addition to potential exaggerated immune-induced diseases [14]. The lack of association between the two cytokines and the BMI and age in the current study implies that reducing Pb exposure could mitigate measures against the low levels of circulating cytokines observed in subjects with elevated BLLs. Moreover, sensitization efforts targeted at lowering the BLLs among women of reproductive age in Kabwe, which were higher than the levels reported in adults living near a mining site in South Africa [28] and comparable to the overall weighted mean BLLs reported in pregnant women in Sub-Saharan Africa [29], must be heightened.

This study has some limitations. The first limitation was the small sample size, although the selection represented a broad section of Kabwe residents. The lack of other analyses on the general blood and hormonal profiles is another limitation. This being a cross-section study naturally entails that follow-up studies must be conducted to further explore the impact of chronic environmental Pb exposure with a large sample size. Notwithstanding, the study has established an association between chronic environmental Pb exposure and some plasma cytokines. We, therefore, recommend that further studies be conducted to ascertain the impact of the long-standing environmental Pb pollution on plasma cytokines and other immune mediators in adult residents in Kabwe, Zambia.

## 5. Conclusions

Chronic environmental lead exposure as it has been ascertained in Kabwe, Zambia, could be associated with negative effects on the levels of the circulating plasma TNF-α and IL-8 cytokines in adult female and male subjects. The female subjects showed increased levels of TNF-α in the low blood lead level group and a reduction in the TNF-α levels in the high blood lead level group. In male subjects, the impact of lead exposure on the levels of TNF-α and IL-8 was not significant between the low blood and high blood lead level groups. A negative correlation between BLLs and TNF-α was found, suggesting that an increase in BLLs could lead to a reduction in TNF-α and IL-8 levels. Therefore, our findings suggest that adult females under Pb exposure may be more vulnerable and susceptible to TNF-α and IL-8 dysregulation-linked diseases than their male counterparts. Further studies are recommended to investigate the impact arising from the effect of lead on immunomodulatory cytokines, especially in females.

## Figures and Tables

**Table 1:** General characteristics of adult female and male subjects sampled.

**Gender: Female**	**Low BLL (*n* = 47)** **Mean ± SD (Min–Max)**	**High BLL (*n* = 21)** **Mean ± SD (Min–Max)**	***p*-Values**
BLL (µg/dL)	3.76 ± 2.21 (0.79–7.59)	23.5 ± 7.95 (14.1–42.5)	<0.001 *
Age (years)	37.7 ± 12.3 (27–69)	36.5 ± 14.6 (21–68)	0.511
BMI (kg/m^2^)	24.6 ± 5.23 (15.2–37.9)	24.1 ± 4.07 (18.4–31.9)	0.695
Smoking status	Never smoked	Never smoked	
**Gender: Male**	**Low BLL (*n* = 43)** **Mean ± SD (Min–Max)**	**High BLL (*n* = 18)** **Mean ± SD (Min–Max)**	***p*-Values**
BLL (µg/dL)	4.13 ± 2.12 (1.18–7.96)	23.7 ± 7.28 (15.5–40.7)	<0.001 *
Age (years)	39.3 ± 13.4 (19–72)	47.7 ± 12.5 (21–66)	0.008 *
BMI (kg/m^2^)	24.1 ± 5.03 (18.6–38)	(21.9 ± 3.00 (16.2–29.7)	0.293
Smoking status	Never smoked	Never smoked	

Mann–Whitney U test (*p* < 0.05, * represents statistical significance). BMI (body mass index), BLL (blood lead level).

**Table 2:** Correlations of cytokines and independent variables in females (*n* = 68).

Variables	BMI	Age	IL-8	TNF-α	BLL
BMI (kg/m^2^)				-	-
Age (years)	0.322 *				
IL-8 (pg/mL)	−0.091	0.084			
TNF-α (pg/mL)	−0.104	0.061	0.288 *		-
BLL (µg/dL)	−0.145	−0.183	−0.028	−0.150	

* Spearman’s correlation coefficient *p* < 0.05. BMI (body mass index), IL-8 (interleukin-8), TNF-α (tumor necrosis factor alpha), BLL (blood lead level).

**Table 3:** Correlations of cytokines and independent variables in males (*n* = 61).

Variables	BMI	Age	IL-8	TNF-α	BLL
BMI (kg/m^2^)				-	-
Age (years)	0.172				
IL-8 (pg/mL)	−0.048	−0.069			
TNF-α (pg/mL)	−0.035	−0.075	0.163		-
BLL (µg/dL)	−0.137	0.372 *	0.050	−0.133	

* Spearman’s correlation coefficient *p* < 0.05. BMI (body mass index), IL-8 (interleukin-8), TNF-α (tumor necrosis factor alpha), BLL (blood lead level).

## Data Availability

Data is unavailable due to privacy and ethical restrictions.

## Supplementary Materials

The following supporting information can be downloaded at: https://www.mdpi.com/article/10.3390/ijerph20085596/s1, Table S1: Loading matrix for the PCA analysis, Table S2: Correlations estimated by a Row-wise Method.
