# Supplementary material for: Association between Chronic Environmental Lead (Pb) Exposure and Cytokines in Males and Females of Reproductive Age from Kabwe, Zambia
Source: Int J Environ Res Public Health. 2023 Apr 20;20(8):5596. doi: 10.3390/ijerph20085596 (PMC10138795; doi:10.3390/ijerph20085596)
Supplement: Supplementary file 1 [file ijerph-20-05596-s001.zip › ijerph-2262727-supplementary.pdf]

*Supplementary Materials*

# Association between Chronic Environmental Lead (Pb) Exposure and Cytokines in Males and Females of Reproductive Age from Kabwe, Zambia

**Andrew Kataba [1,2], Yared Beyene Yohannes [1,3], Hokuto Nakata [1], John Yabe [2,4], Haruya Toyomaki [1], Kaampwe Muzandu [2], Golden Zyambo [2], Yoshinori Ikenaka [1,5,6,7], Kennedy Choongo [2], Mayumi Ishizuka [1,*] and Shouta M. M. Nakayama [1,2,*]**

[1] Laboratory of Toxicology, Department of Environmental Veterinary Sciences, Faculty of Veterinary Medicine, Hokkaido University, Kita 18 Nishi 9, Kita-ku, Sapporo 060-0818, Japan
[2] School of Veterinary Medicine, The University of Zambia, Lusaka P.O. Box 32379, Zambia
[3] Department of Chemistry, College of Natural and Computational Science, University of Gondar, Gondar P.O. Box 196, Ethiopia
[4] School of Veterinary Medicine, University of Namibia, Windhoek P/B. 13301, Namibia
[5] Water Research Group, School of Environmental Sciences and Development, North-West University, Potchefstroom 2531, South Africa
[6] Translational Research Unit, Veterinary Teaching Hospital, Faculty of Veterinary Medicine, Hokkaido University, Sapporo 060-0818, Japan
[7] One Health Research Center, Hokkaido University, Sapporo 060-0818, Japan
* Correspondence: ishizum@vetmed.hokudai.ac.jp (M.I.); shoutanakayama0219@gmail.com or shouta-nakayama@vetmed.hokudai.ac.jp (S.M.M.N.); Tel.: +81-11-706-6949 (M.I.); +81-11-706-5105 (S.M.M.N.); Fax: +81-11-706-5105 (M.I.& S.M.M.N.)

The supplementary data as contained in Tables S1 and S2 shows the loading matrix for the Principal Component Analysis (PCA) and the Correlations as estimated by the Row-wise method, respectively.

**Table S1.** Loading matrix for the PCA analysis.

| Variable | PC1 (27.6%) | PC2 (24.4%) |
|---|---|---|
| Age (yrs) | **0.447** | **0.526** |
| BLL (µg/dL) | **0.486** | **-0.518** |
| TNF-α (pg/mL) | **-0.680** | 0.323 |
| IL-8 (pg/mL) | **-0.585** | 0.075 |
| BMI (kg/m$^2$) | 0.370 | **0.758** |

BMI (Body Mass Index), IL-8 (Interleukin-8), TNF-α (Tumor necrosis factor alpha), BLL (Blood Lead Level). PC1 (Principal component 1), PC2 (Principal component 2). The values in bold represent variables with significant contributions to the respective principal components.

**Table S2.** Correlations estimated by a Row-wise Method.

| Variables | Age (yrs) | BLL (μg/dL) | TNF-$\alpha$ (pg/mL) | IL-8 (pg/mL) | BMI (kg/m2) |
|---|---|---|---|---|---|
| Age | 1.000 | 0.093 | -0.052 | 0.003 | **0.244** |
| BLL | 0.091 | 1.000 | **-0.210** | -0.078 | -0.124 |
| TNF-$\alpha$ | -0.052 | **-0.210** | 1.000 | **0.206** | -0.030 |
| IL-8 | 0.003 | -0.078 | **0.206** | 1.000 | -0.121 |
| BMI | **0.244** | -0.124 | -0.021 | -0.121 | 1.000 |

BMI (Body Mass Index), IL-8 (Interleukin-8), TNF-$\alpha$ (Tumor necrosis factor alpha), BLL (Blood Lead Level). The values in bold represent variables with significant correlation estimated by the Row-wise method.
